# Comparison of Thermal Neutron and Hard X-ray Dark-Field Tomography

**DOI:** 10.3390/jimaging7010001

**Published:** 2020-12-23

**Authors:** Alex Gustschin, Tobias Neuwirth, Alexander Backs, Manuel Viermetz, Nikolai Gustschin, Michael Schulz, Franz Pfeiffer

**Affiliations:** 1 Chair of Biomedical Physics, Department of Physics and Munich School of Bioengineering, Technical University of Munich, 85748 Garching, Germany; manuel.viermetz@tum.de (M.V.); nikolai.gustschin@tum.de (N.G.); franz.pfeiffer@tum.de (F.P.); 2 Heinz Maier-Leibnitz Zentrum (MLZ), Technical University of Munich, 85748 Garching, Germany; Tobias.Neuwirth@frm2.tum.de (T.N.); Alexander.Backs@frm2.tum.de (A.B.); Michael.Schulz@frm2.tum.de (M.S.); 3 Physik-Department E21, Technical University of Munich, 85748 Garching, Germany; 4 Department of Diagnostic and Interventional Radiology, Klinikum Rechts der Isar, Technical University of Munich, 81675 Munich, Germany

**Keywords:** neutron dark-field imaging, neutron grating interferometry, neutron imaging, X-ray grating interferometry, hard X-ray dark-field imaging

## Abstract

High visibility (0.56) neutron-based multi-modal imaging with a Talbot–Lau interferometer at a wavelength of 1.6 Å is reported. A tomography scan of a strongly absorbing quartz geode sample was performed with both the neutron and an X-ray grating interferometer (70 kVp) for a quantitative comparison. Small scattering structures embedded in the absorbing silica matrix were well resolved in neutron dark-field CT slices with a spatial resolution of about 300 μm. Beneficial effects, such as monochromaticity and stronger penetration power of the used neutron radiation, helped to avoid the beam hardening-related artificial dark-field signal which was present in the X-ray data. Both dark-field modalities show mostly the same structures; however, some scattering features appear only in the neutron domain. Potential applications of combined X-ray and neutron multi-modal CT enabling one to probe both the nuclear and the electron density-related structural properties are discussed. strongly absorbing samples are now accessible for the dark-field modality by the use of thermal neutrons.

## 1. Introduction

Computed tomography (CT) is a versatile and non-destructive imaging method used with different kinds of radiation to yield three-dimensional (3D) representations of complex objects which are often not accessible with other methods. It usually uses projectional images or signals which are created by physical interactions inside the object to reconstruct a 3D distribution of some physical property. Different types of CT can be combined for special applications to study different physical interactions in correlation to their spatial distribution. Many applications have been developed to exploit complementary tomographic methods, such as X-ray CT and positron emission tomography, where radioactive tracers are used to mark certain molecular processes in the human body and create overlays of, e.g., metabolic tumor activity in an X-ray CT image. Another example is the combination of conventional CT with X-ray fluorescence analysis in scanning mode, enabling one to detect spatial distributions of sub-parts-per-million trace elements in transmission tomograms which would never be possible by the absorption mechanism only.

A recently emerging method is combined neutron and X-ray CT, where two different kinds of interactions are used to correlate nuclear and electron density-related properties of a sample spatially. X-rays interact with the atomic electron shells and therefore have a continuously increasing absorption cross-section with the atomic number and depend on the over-all electron density distribution in the object. Neutrons, however, mostly interact with the nuclei of the material due to their neutral charge. Therefore, they can even generate contrast between different isotopes of the same element. Compared to X-rays, they easily penetrate many heavy elements, such as gold, tungsten and lead, but are strongly absorbed or scattered by, e.g., hydrogen and therefore generate contrast with many organic materials. Such combined X-ray and neutron imaging systems have been implemented at the Institut Laue-Langevin (ILL) in Grenoble, France [1], the Paul Scherrer Institute (PSI) in Villingen, Switzerland [2] and the National Institute of Standards and Technology (NIST), Gaithersburg, USA [3]. This dual imaging approach was used for many sample-systems, such as fuel cells [4], batteries [5], geosciences [6], building materials [7], cultural heritage objects [8] and others. In the last two decades, even more types of interactions became accessible with X-rays and neutrons sensing the refractive and dispersive properties of materials with X-ray grating interferometry (XGI) [9,10,11] and neutron grating interferometry (nGI) [12,13]. Neutron dark-field imaging is uniquely sensitive to magnetic domains [14,15], vortex lattice domain structures in superconductors [16] and micro-porous materials, and could be successfully extended to CT [14,15]. Such imaging setups are composed of several diffractive gratings [17] to create fine intensity modulations on the micrometer scale which are then refracted or scattered by the sample. These changes—which are smaller in nature than the resolution of the system—can be sensed by a sampling process with an absorption grating in front of the detector and transferred into an image. For that, one of the gratings is mechanically stepped in the range of one period and an image of the interferogram is recorded at each step. The procedure is then repeated without the sample in the beam path. Typically, this results in two sinusoidal modulations in every pixel which are shifted in phase and altered in amplitude and mean values. From that, three images can be calculated: The quotient of the mean intensity values $T=I/I_{0}$ delivers an attenuation image which is analog to a conventional radiograph. The phase shifts give a differential phase-contrast (DPC) image which highlights refractive gradients and can be useful in many cases where absorption does not create enough contrast. The relative reduction of the amplitude provides a dark-field image (DFI) which reveals structures that exhibit small-angle scattering (SAS) [11]. The dark-field signal is defined by [18]:

(1) $$D=V/V_{0},$$

where *V* is the visibility calculated from the maximum ($I_{max}$) and minimum ($I_{min}$) of the fitted sinusoidal intensity modulation in every pixel by:

(2) $$V=\frac{I_{max}-I_{min}}{I_{max}+I_{min}}.$$

$V_{0}$ is the visibility without the sample often referred to as the flat field visibility. Using the principle of a Talbot–Lau interferometer, this can also be realized with radiation sources of low coherence, such as conventional X-ray tubes [12] or neutron imaging beam lines with relatively large pinholes that are crucial for reasonable exposure times [10].

This method has certain limitations—especially with strongly absorbing objects. If a high fraction of the radiation is absorbed along a certain beam path through the object, the scattering and phase signals are affected by strong noise. Further, such interferometers are chromatic in nature and therefore create an energy-dependent visibility. When polychromatic radiation sources are used, the energy-dependent interaction will introduce artifacts in the DPC and the DFI [19,20]. The DFI is especially affected by a disproportional amplitude reduction because of beam hardening and will generate additional signal by purely absorbing objects [20]. Several correction approaches have been put forward [19,20,21]; however, it remains a challenge, especially due to the limited statistics when highly absorbing objects are investigated. There are also achromatic realizations of DPC and DFI setups, e.g., aperture-based imaging relying purely on geometric beam modulations [22]. Those, however, mostly operate with sources of high coherence using very small focal points or pinholes in the case of neutron imaging and require high resolution detectors [23] or detector masks [24], which reduces their efficiency. Even using an achromatic system, the refractive and scattering behaviors in the sample itself are energy-dependent and complicate obtaining quantitative results with broad spectra.

Hence, the usability of the method strongly depends on the geometrical shape, the chemical composition and particularly the ratio of the absorption to small-angle scattering cross-sections of the involved materials and microstructures in the sample. Studying scattering structures in strongly attenuating objects becomes difficult and often requires higher-energy radiation. The energy, and therefore the penetrative power of such imaging systems, are limited by the absorptive properties of the gratings. X-ray-based Talbot–Lau imaging systems typically do not exceed 50 keV design energy, since the absorptive gratings become transparent to high energy X-rays; the propagation distances to induce the intensity modulation via the Talbot effect become longer; and the refractive and scattering interactions become weaker with shorter wavelengths. All those factors require gratings with smaller periods and greater heights of the absorbing material and become extremely difficult to fabricate. Neutron-based Talbot–Lau imaging so far has used wavelengths of about 3 Å and above due to similar grating-related limitations. We have recently reported on a grating fabrication method [25] which allowed us to improve the performance of the nGI instrument at the ANTARES beamline significantly [26]. In this work, we present neutron-based DFI and DPC imaging at a wavelength of 1.6 Å which coincides with the peak of the ANTARES beamline spectrum at FRM II [27]. A quartz geode containing absorbing and scattering structures was used for demonstration, and an entire grating-based CT was recorded with thermal neutrons and with hard X-rays. Both scans are compared with a focus on the dark-field modality by discussing application cases and the benefits of using neutrons and X-rays complementarily.

## 2. Methods

### 2.1. Neutron Grating Interferometer Setup

**Figure 1:**
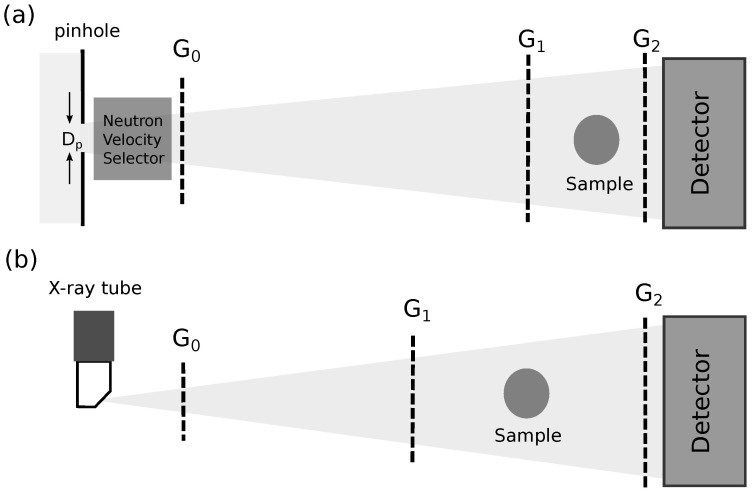
Schematic illustration (not to scale) of the neutron grating interferometer geometry (**a**) compared to the symmetric X-ray grating interferometer (**b**).

The neutron grating interferometer at ANTARES/FRM II was designed in an asymmetric Talbot–Lau geometry finding a trade-off between sensitivity and achievable resolution while using relatively large L/D ratios for a high neutron flux. The geometry and important performance indicators of the instrument were described in a recent work in detail [26]. Here, the focus lies on imaging at a neutron wavelength of 1.6 Å to exploit the high flux of neutrons available in the spectrum at ANTARES and extending the method to study larger and stronger attenuating objects. Figure 1a shows the outline of the imaging system. The neutron radiation leaves the pinhole of $D_{p}=35.6\;\mathrm{mm}$ and is monochromatized by a neutron velocity selector to a bandwidth of Δλ/λ=20%. The source grating with a period of $p_{0}=150\;\mu m$ and a duty cycle (fraction of transparent area) of 0.3 creates an array of line sources providing enough spatial coherence for a good contrast of the interferometer. The phase grating $G_{1}$ has a period of 24.4 μm, a duty cycle of 0.55 and consists of a binary profile of ∼ 123 μm silicon introducing a neutron phase shift of ∼π. It creates an intensity modulation with doubled frequency 60.9 cm downstream from $G_{1}$. It is analyzed with another absorption grating $G_{2}$ which has a period of 13.3 μm and a duty cycle of 0.45. The rotation center of the sample was 55.8 cm downstream from $G_{1}$, and the field-of-view (FoV) was limited to 71 mm×76 mm by the detector. To evaluate a possible application of a strongly attenuating object, a quartz geode was chosen featuring grown silica crystals in the inner cavity. The detection system consisted of an Andor Neo CMOS camera coupled optically to a 100 μm LiF/ZnS scintillation screen and resulted in an effective pixel size of 33 μm (66 μm after binning for reconstruction). For every phase step, 3 frames with 5 s exposure time each were acquired to remove complex noise structures, such as gamma spots [28,29]. A phase scan of every projection consisted of 8 phase steps, and in total 360 views were collected over a 180 ${}^{{}^{\circ}}$ sample rotation. Every 20 projections, the sample was moved out of the FoV and a set of flat-fields was acquired. The flat-field visibility was ∼0.56.

### 2.2. X-ray Grating Interferometer Setup

The X-ray scans were performed with a symmetric Talbot–Lau interferometer illustrated in Figure 1b. The X-ray microfocus tube (XWT-160-CT, X-rayWorX, Garbsen, Germany) with a tungsten reflection target was operated at 70 kVp tube voltage and the beam was filtered by 4 mm aluminum to yield hard X-rays with enough penetrative power for the quartz geode sample. All three gratings used for this work had a period of 10 μm. The source grating $G_{0}$ and the analyzer grating $G_{2}$ were absorption gratings with an absorbing gold height of >170 μm. The phase grating had a binary profile etched in silicon with a height of 60 μm and was designed to introduce a π-shift for the spectral range around 47 keV. The inter-grating distance was 90.0 cm, and the sample rotation center was 64.0 cm from $G_{1}$ to fit into the FoV limited by the size of the $G_{2}$ grating (70 mm×55 mm). A Varex XRD 4343CT detector with a pixel size of 150 μm was operated at an exposure time of 2 s per frame. The effective pixel size in the reconstructed volume was 118 μm. Over the whole FoV, a flat field visibility of ∼0.24 was reached. Every phase scan consisted of 11 steps, and in total 361 projections were acquired. Flat fields were collected only once prior to the scan.

### 2.3. X-ray Micro-CT Scan

On top of the grating-based multimodal data, the sample was scanned with a conventional X-ray micro–computed tomography (micro-CT) system (GE phoenix v|tome|x S) with a high resolution at 130 kVp tube voltage and 0.6 mm copper filtration. This energy range is currently not available with XGI at reasonable operating parameters, since the mean spectral energy is too high for state-of-the-art absorption gratings. The effective pixel size was 70 μm and in total 1601 projections were collected over 360 ${}^{{}^{\circ}}$ sample rotation. The exposure time per frame was 6 s.

### 2.4. Data Processing

**Figure 2:**
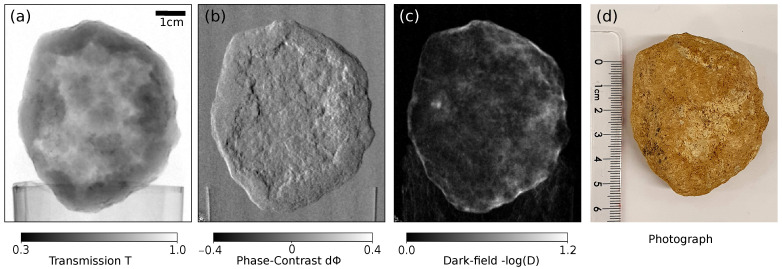
(**a**–**c**) One projection of the quartz geode in the three channels acquired at 1.6 Å neutron wavelength. Attenuation image (**a**), differential phase-contrast image (**b**), dark-field image (**c**) and a photograph (**d**).

In the case of neutron data, the acquired images were corrected for dark current with averaged dark frames, and defective pixels were interpolated by a median with a kernel size of 3. Afterwards, artificial bright structures (gamma spots) characteristic for neutron scintillator-based CCD detectors were removed by a slightly adapted algorithm compared to the standard procedure [28,29] described in the following. For every frame, a median-filtered image with a kernel size of 9 pixels was calculated and subtracted from the original image. From that a binary mask labeling the artificial features was created by a threshold manually chosen for the respective exposure levels and density of the spots. Finally, all three original images were averaged weighted by the respective masks. Pixels, which were affected by the noise in all three images, were interpolated by a median of the resulting image with a kernel size of 3. Compared to a simple median average over all three images, this approach delivers better results on pixels which are affected by the noise in two or even all three frames and therefore can handle frames with high density of gamma spots or when only 2 images are available. At the same time, nearly all features and edges remain preserved and much less pixels have to be interpolated in the present example. After the denoising, the neutron images were binned by a factor of 2 in both directions since both the point spread function (PSF) of the detector and the geometrical blur due to the source size were limiting the resolution much more than the pixel size. The phase scans were then processed by an expectation maximization algorithm which minimizes the residuum iteratively taking into account that the grating positions during stepping were not precisely equidistant and the overall stepping range could deviate from one period due to experimental deficiencies [30]. The attenuation, differential phase-contrast and the dark-field images were calculated with the processed flat fields. One projection is depicted in Figure 2 with all three modalities and a photograph of the sample.

**Figure 3:**
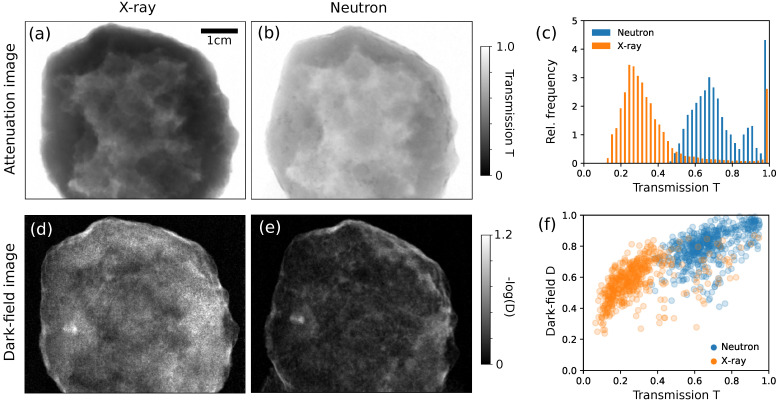
Quantitative comparison of an angular projection of the quartz geode. X-ray attenuation image (**a**) at 70 kVp, neutron attenuation image (**b**) at 1.6 Å and histogram of the grey values of both images (**c**). X-ray dark-field image (**d**), neutron dark-field image (**e**) and a scatter plot (**f**) showing the ratio of attenuation and the dark-field related visibility loss of 500 random pixels from all projections.

The X-ray data were processed with the same algorithm after the dark current correction and bad pixel masking without any further image filtration. The projections and flat fields were normalized using a sample-free background to correct for flux fluctuation of the X-ray source. Figure 3 shows a conventional attenuation image (a) and an X-ray DFI (d) of the sample at a view angle where the geode is flat and the total transmittance is relatively high. As a comparison, roughly the same projection in the neutron attenuation (b) and dark-field channel (e) is shown at the same gray levels as the X-ray data. The histogram in Figure 3c shows the distribution of attenuation values in the X-ray and the neutron attenuation image. Figure 3f illustrates the ratio of the attenuation signal $T=I/I_{0}$ and the dark-field-related visibility loss $D=V/V_{0}$ extracted from 500 random pixels from all X-ray and neutron projections inside the sample. Note that the images do not show the entire sample, since it did not fit into the FoV of the X-ray interferometer, and therefore the X-ray data do not contain the entire object.

**Figure 4:**
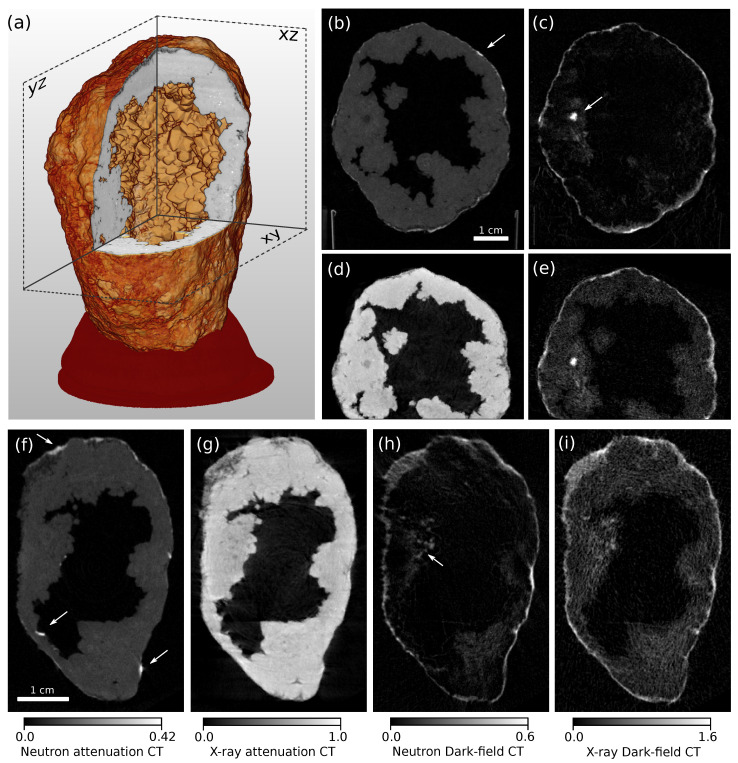
(**a**) Rendering of the X-ray micro-CT reconstruction with an incision to illustrate the hollow inner structure of the quartz geode. Slices from the filtered back-projection (FBP) (**b**–**e**) in the yz-plane showing the neutron attenuation (**b**), neutron dark-field (**c**), X-ray attenuation (**d**) and X-ray dark-field (**e**) channels. Slices (**f**–**i**) in the xy-plane depicting the neutron attenuation (**f**), X-ray attenuation (**g**), neutron dark-field (**h**) and X-ray dark-field (**i**) channels. The color bars in (**f**–**i**) apply to the respective slices in (**b**–**e**) as well, and the upper boundaries correspond to maximal values of the FBP found in the respective slices.

**Figure 5:**
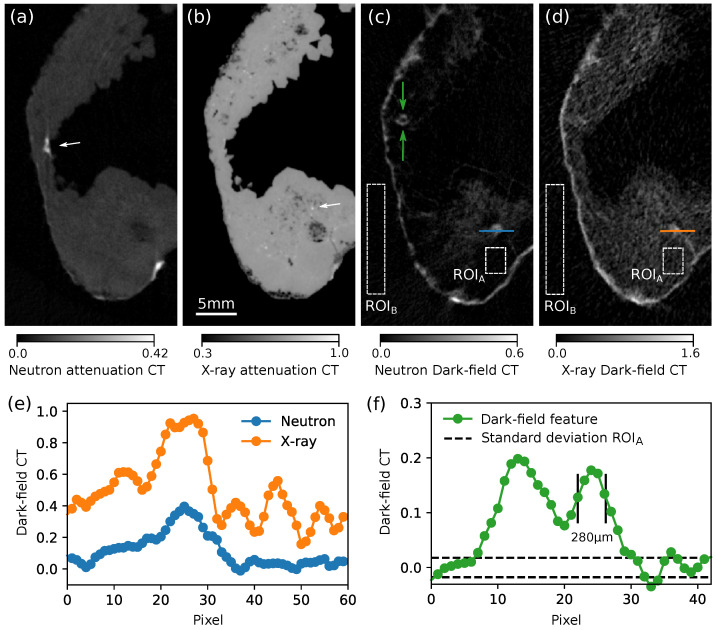
Slices in the xy-plane from the neutron attenuation volume (**a**), X-ray micro-CT (**b**), neutron DFI (**c**) and X-ray DFI (**d**). A line plot (see lines in (**c**,**d**) through a scattering feature which is used for CNR calculation is shown in (**e**). Another line plot (**f**) of the structure marked by green arrows in (**c**) plotted compared to the noise level (upper and lower boundaries of the standard deviation of ROI_A_).

The tomographic reconstruction consisted of a filtered back-projection (FBP) of −log(T) for the attenuation and −log(D) for the dark-field domain with a Ram-Lak filter. It was performed together with a center shift correction and a ring artifact removal algorithm provided by the software X-Aid FDK Reconstruction Suite 2020.10.2 (Mitos GmbH, Garching, Germany). The absolute values of the FBPs (used in the figures as units for the CT slices) are proportional to the effective linear attenuation coefficient $\mu_{eff}$ for the attenuation CT and the linear diffusion coefficient (sometimes also called dark-field extinction coefficient) ϵ [31,32] for the dark-field CT. Finally, the X-ray volume was digitally registered to the neutron data using the software Avizo Fire 8.1.0. Figure 4 shows a 3D rendering (a) and horizontal (b-e) and vertical (f-i) slices of the reconstructed volume in all four image domains. The upper limits of the gray scale correspond to the maximal absolute CT values of the respective slices. Figure 5 shows slices from the xy-plane of the sample through the scattering structure depicted by an arrow in Figure 4c. Figure 5a shows the slice from the neutron attenuation CT; (b) shows the high resolution micro-CT scan; (c) shows the neutron DFI; (d) the X-ray DFI and line plots from some scattering features are shown in (e) and (f). To compare the contrasts achieved by neutrons and X-rays, suitable ROIs were chosen: to evaluate the background DFI signal, ROI_B_ (outside the sample); and ROI_A_ (inside the sample) where only absorbing material is present. The contrast-to-noise-ratio (CNR) was retrieved from the peak signal of the scattering structure $S_{S}$ shown in the line plots (Figure 5e), the background $S_{B}$ in the sample (ROI_B_) and the standard deviation $\sigma_{B}$ in ROI_B_ with the formula:

(3) $$CNR=\frac{S_{S}-S_{B}}{\sigma_{B}}.$$

The mean values and standard deviations of the ROIs and the CNRs are given in Table 1.

## 3. Results and Discussion

When evaluating the projections shown in Figure 3, it becomes apparent that the neutron imaging setup has a significantly higher penetration power for the sample consisting mostly of silicon oxide crystals. The images show an angular view where the sample is relatively flat yielding maximal transmission values. The histogram in Figure 3c shows that the neutron transmission values are more than twice as big as the X-ray values, which go below 0.3 for a high portion of the data. Hence, pronounced beam hardening artifacts and strong noise in the dark-field channel can be expected in X-ray CT. A comparison between the dark-field images (d) and X-ray DFI (e) shows that similar scattering structures and edges are resolved. However, the X-ray DFI suffers from significantly higher noise as expected due to stronger absorption of the X-ray radiation. Further, an outline of the absorbing structure visible in the attenuation image (Figure 3a) is also recognizable in the DFI (d) due to the beam hardening effect discussed above. The noise level in the neutron DFI (e) is significantly lower compared to the X-ray DFI (d) and the visibility loss as seen from (f). That means that the neutron DFI system has a significantly wider dynamic range to even cope with larger and stronger scattering samples. Note also that the angular sensitivity in the neutron scan is significantly lower, since the sample is much closer to the $G_{2}$ than in case of the X-ray scan. Therefore, it would even be possible to reach a better sensitivity in the nGI and generate even more contrast in the neutron DFI by placing the sample closer to the $G_{1}$. However, this would come at the cost of image resolution due to the geometric blur by the neutron source.

The tomographic reconstruction of the different imaging modalities reveals further differences that were not obvious from projectional data. Figure 4 shows a rendering of the sample generated from the micro-CT data and slices from two different planes in all four image channels. An incision visualizes the inner cavity of the quartz geode covered with silica crystals. The slices from the neutron attenuation data (b) and (f) show significantly lower values than the respective X-ray slices (d) and (g). Note here that the absolute gray values from the neutron attenuation CT slices are even windowed much narrower than those of the X-ray CT slices. Further, strongly absorbing structures appear in the neutron data (arrows in (b) and (f)) that are not recognizable at all in the respective X-ray slices. The opposite is visible in Figure 5a,b, where the X-ray slice (b) shows small absorbing grains (see arrow) which are not visible in the neutron attenuation image (a). However, in (a) highly absorbing material (see arrow) is present that is not visible in (b). For further investigation, some of this highly neutron-absorbing material located on the surface of the sample was extracted and analyzed by X-ray fluorescence. On top of silicon (Si), significant amounts of calcium (Ca) and chlorine (Cl) and rather small amounts of sulfur (S), phosphorus (P), potassium (K) and iron (Fe) were confirmed by $K_{\alpha }$ fluorescent lines. Most likely the bright structures visible in the neutron tomograms were some minerals of Ca and Cl, since they attenuate significantly more strongly than Si. In the X-ray domain, however, a much smaller contrast to Si is expected between those elements. Since Fe was present in the mineral composition of the sample, it might have been the X-ray absorbing element causing the bright grains (see arrow) in Figure 5b. Combining both X-ray and neutron scans, additional elements or compounds can be detected or excluded by complementary data, as this example shows.

The dark-field CT data emphasizes the findings that could be already vaguely concluded from single projections. A comparison of the slices from Figure 4c,h to the respective X-ray dark-field slices (e) and (i) again shows that bulk attenuating material causes artificially low frequency noise in the X-ray DFI, and makes small scattering structures such as those depicted by the arrow in (h) hardly recognizable in the respective X-ray DFI. The structures depicted there have sizes of about 300 μm and can be well resolved and differentiated from the noise in the background. Figure 5 shows a magnified view from a slice in the xy-plane where the micro-CT image (b) reveals porous structures around the big scattering feature that is well visible in both dark-field channels (c,d). Those structures are recognizable in the neutron DFI (c); however, they are completely overlaid by the artificial dark-field signal in the respective X-ray DFI (d). Further, a scattering feature depicted in (c) by green arrows is not visible in the respective X-ray DFI at all. It seems that this structure is composed of different material than the scattering compound covering the outer surface of the sample, since the latter also generates a strong signal in the X-ray domain. This can be explained by the presence of some chemical compound involving elements with nuclei that have strong nuclear small-angle scattering cross-sections but no electron density modulation on the micron scale that would create significant DFI signals with X-rays. Hence, neutron DFI might detect additional structures that hardly scatter X-rays and deliver additional elemental and magnetic sensitivity. The reverse case, features that are present in the X-ray DFI but not in the neutron DFI, could not be identified with certainty, since low frequency noise structures could be mistaken for real scattering features in the X-ray DFI data. Note that the objective of this work was not to identify the scattering compounds but to show that scattering features can be resolved despite being embedded in strongly absorbing surroundings.

To quantify the artificial, absorption-induced dark-field signal in both DFI CT reconstructions, the FBP values (mean and standard deviation) of some ROIs inside of absorbing material (ROI_A_) and outside the sample (ROI_B_) were calculated and given in Table 1. Note that ROI_A_ was chosen inside the sample where the X-ray micro-CT data do not show any structures that could potentially create SAS signals. While there is a strong signal increase (both mean value and standard deviation) in the case of X-rays, the neutron DFI signal hardly increases and the beam hardening-related artifacts do not deteriorate the image. Note that even the strongly absorbing structure depicted by the arrow in Figure 5a does not create any signal in the respective neutron DFI (c). Figure 5e shows a line plot comparison of a scattering feature (see blue and orange lines in (c) and (d)) which was used for a CNR estimation. Although the X-ray signal is more than two times stronger, it is much less apparent in the surrounding noise. Its CNR in the neutron DFI is more than twice as big compared to the X-ray DFI, as the values from Table 1 show. A line plot of the small scattering structure (which is not visible in the X-ray DFI) depicted by the green arrows in Figure 5c is given in (f) and shows that a feature of about 280 μm can be well resolved with a CNR of ≈ 7.5 in the neutron DFI modality. The resolution of the nGI CT scan was limited by the number of angular projections which could be acquired during a measurement time of about 13 h. A higher resolution and better image quality can be achieved with more projections. To go beyond 200 μm, the source size defined by the pinhole diameter $D_{p}$ has to be decreased, which would increase the measurement time drastically. To go even beyond 100 μm, a different scintillator with a lower efficiency would be required coming at the cost of even longer exposure times. The resolution of the XGI CT scan was limited by the PSF of the X-ray flat panel detector. A higher resolution could be reached by scanning the sample with a higher magnification by placing it closer to or even in front of the $G_{1}$. However, the FoV due to the grating sizes would be too small to cover the full width of the sample and would require stitching.

## 4. Conclusions

Neutron-based imaging with a Talbot–Lau interferometer has been successfully translated to sub- 2 Å wavelengths and can be extended to a larger variety of strongly absorbing samples. Compared to other recent works, the setup reached high visibility, high neutron flux and a relatively large FoV. The method was also demonstrated in CT, with the attenuation and the dark-field signal reaching a resolution of about 300 μm. To the best of our knowledge, the present work is the first to acquire an attenuation and a dark-field CT with both thermal neutrons and hard X-rays on the same sample. As the studied example shows, the dark-field signal is difficult to access when the sample consists of strongly absorbing structures. Especially if the structures of interest are rather small and embedded in the absorbing material, the artificial dark-field signal related to beam hardening will introduce additional background noise in radiography and CT, and will deteriorate the resolution. Here, nGI benefits from quasi-monochromatic filtration that is possible with neutrons and from a higher penetration power for many materials compared to current X-ray grating interferometry. Not only for geological specimens, but also for sample systems or environments where, e.g., heavy metals are involved and additional contrast between organic compounds, magnetic materials or even isotopes is of interest, nGI might deliver unique complementary insights. Conventional X-ray micro-CT, on the other hand, benefits from higher flux with small source sizes and is therefore able to provide a high spatial resolution.

The scans in this work have been performed with two different setups. To simplify alignment issues and be able to study processes in situ without temporal separation using both neutrons and X-rays conducting simultaneous scans would be of great benefit. Hence, the ANTARES beamline will be extended with a micro-focus X-ray source and a high resolution detector to acquire micro-CT and neutron data at the same time. Additionally, this can be combined with different sample environments that are usually not available in conventional micro-CT laboratories. This complementary CT approach with X-rays and multi-modal nGI will deliver further detailed insights for a wider variety of especially strongly absorbing samples with additional sensitivity to many different elements or chemical compounds. Complex geometries with unique micromorphologies, such as porous structures or magnetic domains, will be better accessible non-destructively in 3D.

## Figures and Tables

**Table 1:** Comparison of X-ray and neutron DFI signals and CNRs of a scattering feature shown in Figure 5c,d.

Signal	Background ROI_B_	Absorber ROI_A_	Signal *S_S_* of SAS Feature	CNR
X-ray DFI	−0.0016 ± 0.0471	−0.2338 ± 0.1016	0.96	7.1
Neutron DFI	−0.0005 ± 0.0176	−0.0072 ± 0.0228	0.39	16.8

## Data Availability

The data presented in this study are available on request from the corresponding author.

## Abbreviations

The following abbreviations are used in this manuscript:

CT	computed tomography
micro-CT	micro–computed tomography
3D	three-dimensional
XGI	X-ray grating interferometry
nGI	neutron grating interferometry
DPC	differential phase-contrast
DFI	dark-field image
FoV	field-of-view
ROI	region of interest
SAS	small-angle scattering
CNR	contrast-to-noise-ratio
PSF	point spread function
FBP	filtered-Back Projection
